# Reductio Ad Absurdum

**Published:** 1909-11-06

**Authors:** 


### The Hospital

A Journal of

The Medical Sciences and Hospital Administration.

New Series. No. 141, Vol. VI. [No. 1213, Vol. XLVII.] SATURDAY, NOVEMBER 6, 1909.

### Reductio ad Absurdum.

At the risk of wearying our readers and of advertising practices and doctrines which we believe to be morally and physically dangerous, we feel constrained to draw attention to some particular points in the tenets of "Christian Science" as disclosed in the evidence given before the Coroner for Paddington at an inquest held on October 29. Those who saw the report of that inquest may remember that on the morning of the day on which the patient died a medical practitioner was called in by the (unqualified) widow in charge of the case, and that this doctor was asked for a diagnosis, which he furnished. He is reported to have said in his evidence that he ordered no treatment, as he was asked not to do so; but merely examined the dying man with a view to diagnosing the cause of his condition. That a member of our profession should consent to adopt such an attitude of impotent and humiliating inaction seems, in our judgment, too serious a matter to be allowed to pass without protest. It is true that the medical man in question recognised the fact that the unfortunate colonel was *in extremis* and was beyond medical aid. To state frankly that nothing can be done in such a case is obviously a doctor's duty, rather than to delude with false hopes; but in this case the doctor seems to have entered the house on the understanding that diagnosis alone was required of him, and that treatment by the widow lady would be continued. It would be interesting to hear whether this act does or does not constitute "covering" as understood by the General Medical Council, and it may be suggested that the subject might profitably occupy some attention at the next session of that body.

Turning to a more important aspect of this extraordinary case, it may be well to emphasise some admissions obtained from the "Christian Science" practitioner, since they constitute evidence which it does not require a professional training or anything but ordinary common sense to comprehend. The diagnosis of the disease was asked for, not because the patient or his advisers wanted to know it, but because some of his relatives were sceptical as to the efficacy of the "treatment" he was receiving. It is difficult to resist the inference that it was intended to delude these relatives into the idea that skilled medical attention was to be given; for they would naturally suppose that no doctor would undertake to see the patient unless he was to have control of the measures taken for his relief. Putting that point aside, however, what is more important to notice is this: that the diagnosis was not required by the unqualified person in attendance, and was apparently of supreme indifference to her. This attitude, however foolish it may seem to the ordinary layman or medical man, is at any rate consistent; for if prayer, and prayer alone, is the therapeutic agent by which recovery from illness is determined, then obviously to suggest to the Almighty the diagnosis of what He is requested to cure is an impertinence hardly distinguishable from blasphemy. Also to spend any time over the endeavour to find out what is the matter is simply wasting precious moments which should be devoted to those prayers which can alone promote the patient's welfare!

The conclusion is, therefore, quite clear that both in theory and practice the Christian Scientist is wholly indifferent to the diagnosis of the ailment or injury he is called in to treat. That the addition of "injury" to this statement is justified, is proved by the evidence given at the inquest by the widow already referred to; for she is reported to have said that a broken leg can be efficiently treated by prayers without medical attention. Once again it must be pointed out that, whatever else may be thought of the statement, it is a strictly logical deduction from the root principles which these people profess. For if diagnosis is immaterial, then clearly no differentiation can be made between illness and injury, which are so inextricably mingled that it is frequently difficult in a given patient to say what is the exact relation between them: and no differentiation can be made between a slight injury, such as a bruise, and a severe injury, such as a broken bone, for we know that on many occasions the determination of the exact nature of an injury is so difficult that it may be only by the help of $x$-rays that it is possible to decide whether a bone has been broken or not. The attention of the intelligent laity may therefore legitimately be drawn to the fact that according to Christian Scientists a broken leg requires no treatment beyond that of prayer. Not even confinement to bed may be supposed to be essential: for prayer ought to be equally efficacious under any of the purely physical circumstances of a patient's environment. It may be assumed also that a compound fracture, with the bone ends protruding through the skin and smeared with mud or dung from the street, is as amenable to "Christian Science" treatment as a simple fracture: at least, it is difficult to see how the inference is to be resisted. Yet it is to be pointed out to the non-medical reader that since the adoption of antiseptic methods in surgery the mortality of compound fractures has dropped from, roughly speaking, forty to about six per cent. Then, again, there is such a thing as hæmorrhage from a severe wound; though perhaps we ought not to make this bold statement without the consent of Mrs. Eddy and company, who may retort that the bystanders are mistaken, and only *think* they are being splashed with blood. However, we shall assume that there is such a thing as dangerous hæmorrhage, because all doctors, and not a few others, are under the same delusion that they have actually seen it. It should not be difficult to get some enthusiastic advocate of Christian Science to consent to have his femoral artery opened with a knife recently smeared in a culture of tetanus bacilli, on the understanding that should he recover without other treatment than prayer some doctor would undergo the same operation conducted aseptically, and followed by immediate ligature of the vessel! We have considered thus far the logical outcome of a strict application of the principles by adherence to which Lieut.-Colonel the Hon. Charles Alexander, in the judgment of Dr. Spilsbury, the pathologist, lost his life. To pursue the *reductio ad absurdum* further would be easy, but it has already been done so admirably by Mr. Stephen Paget in his recent book, that we rest content with once more recommending its perusal to any who are still unconvinced (or who wish to convince others) of the right that is on the side of the medical profession in this question.

